# GRK3 suppresses L-DOPA-induced dyskinesia in the rat model of Parkinson’s disease via its RGS homology domain

**DOI:** 10.1038/srep10920

**Published:** 2015-06-04

**Authors:** Mohamed R. Ahmed, Evgeny Bychkov, Lingyong Li, Vsevolod V. Gurevich, Eugenia V. Gurevich

**Affiliations:** 1 Department of Pharmacology, Vanderbilt University, Nashville, TN 37232.

## Abstract

Degeneration of dopaminergic neurons causes Parkinson’s disease. Dopamine replacement therapy with L-DOPA is the best available treatment. However, patients develop L-DOPA-induced dyskinesia (LID). In the hemiparkinsonian rat, chronic L-DOPA increases rotations and abnormal involuntary movements modeling LID, via supersensitive dopamine receptors. Dopamine receptors are controlled by G protein-coupled receptor kinases (GRKs). Here we demonstrate that LID is attenuated by overexpression of GRK3 in the striatum, whereas knockdown of GRK3 by microRNA exacerbated it. Kinase-dead GRK3 and its separated RGS homology domain (RH) suppressed sensitization to L-DOPA, whereas GRK3 with disabled RH did not. RH alleviated LID without compromising anti-akinetic effect of L-DOPA. RH binds striatal Gq. GRK3, kinase-dead GRK3, and RH inhibited accumulation of ∆FosB, a marker of LID. RH-dead mutant was ineffective, whereas GRK3 knockdown exacerbated ∆FosB accumulation. Our findings reveal a novel mechanism of GRK3 control of the dopamine receptor signaling and the role of Gq in LID.

The striatum receives dense dopaminergic innervation, and striatal neurons express high levels of dopamine (DA) receptors. DA released by midbrain dopaminergic neurons regulates the striatal output and plays an essential role in movement control. Loss of dopaminergic neurons and depletion of DA in the striatum, as it occurs in Parkinson’s disease (PD), leads to the dysfunction of the striatal circuits and motor deficits. The action of DA in the striatum is mediated by DA receptors, with the D1 and D2 subtypes being the most prominent. The parameters of signaling via DA receptors, as of most G protein-coupled receptors (GPCRs), are defined to a significant extent by a conserved desensitization mechanism. Phosphorylation of activated receptor by a G protein-coupled receptor kinase (GRK) is the first rate-limiting step in this process followed by arrestin binding to phosphorylated receptors. Arrestin blocks further G protein activation and initiates receptor internalization^1^. This mechanism ensures appropriate length and intensity of G protein activation by GPCR preventing their overactivity. Arrestin binding to the receptor also initiates another round of signaling independent of G proteins, via scaffolding of signaling proteins on arrestins^2^. Since most GPCRs require phosphorylation by GRKs for high-affinity arrestin binding^3^, GRKs promote arrestin-dependent signaling, in addition to initiating GPCR desensitization towards G proteins^4^,^5^. A distinguishing feature of the GRKs is the presence, in addition to the kinase domain, of the RGS homology (RH) domain^6^. The RGS proteins are known to be critical regulators of GPCR signaling playing important roles in a variety of physiological and pathological processes^7^. Similarly to many other RGS proteins, the RH of GRK2 and GRK3 is capable of binding active GTP-liganded α-subunits of Gq/11 and recruiting them away from effectors, thereby suppressing Gq/11-mediated signaling in phosphorylation-independent manner^8^. Thus, GRK2/3 can suppress GPCR signaling by two independent mechanisms: receptor phosphorylation and scavenging active Gq/11. While the latter mechanism was demonstrated in cultured cells^9^,^10^,^11^,^12^,^13^,^14^,^15^, it was never shown to operate *in vivo*.

Loss of striatal DA in PD causes complex alterations in cellular signaling: numerous pathways in the DA-depleted striatum show exaggerated responses to stimulation by dopaminergic drugs^16^,^17^,^18^,^19^ linked to supersensitivity of D1^20^,^21^ and D2^22^ receptors. However, the mechanisms that maintain the aberrant receptor responses are yet to be fully elucidated. The symptomatic therapy with the DA precursor L-3,4-dihydroxyphenylalanine (L-DOPA), which is very effective initially in reversing akinesia in PD patients, eventually causes L-DOPA-induced dyskinesia (LID), or involuntary aimless movements^23^. Chronic L-DOPA treatment aggravates the signaling abnormalities initially developed in response to the loss of DA^16^,^24^. The mechanism of LID as well as L-DOPA-induced signaling alterations in DA-depleted animals remains poorly understood. Because dyskinetic and antiparkinsonian actions of L-DOPA are so intertwined, developing anti-LID therapy without sacrificing the beneficial effect of the drug has been a challenge. To successfully manage LID, molecular mechanisms regulating signaling via DA receptors under normal and pathological conditions must be unraveled to enable selective targeting of those specifically responsible for LID. Given the position of GRKs in the GPCR signaling cascade, these proteins are likely to play an important role in the deregulation of the DA receptor signaling in PD. However, the specific role of GRK isoforms in regulating dopaminergic signaling in striatal neurons remains poorly defined.

In the hemiparkinsonian rat model of PD, the concentration of GRKs in the dopamine-depleted motor striatum is reduced, and L-DOPA fails to restore GRK levels^19^,^25^,^26^. Among four GRK isoforms expressed in the striatum^27^,^28^, GRK6 and GRK3 showed particularly consistent downregulation across striatal territories^26^, which may contribute to the exaggerated dopaminergic signaling in the DA-depleted brain. We have previously demonstrated that GRK6 overexpressed in DA-depleted striatum via lentiviral delivery alleviates LID in the rat and primate models, presumably by normalizing DA receptor signaling^25^. Here we show that striatal GRK3, similarly to GRK6, controls L-DOPA-induced behavior and LID in hemiparkinsonian rats. In contrast to GRK6, it does so in phosphorylation-independent manner via its RH domain.

## Results

### Overexpression of GRK3 ameliorates, whereas knockdown enhances behavioral sensitization to L-DOPA in 6-OHDA-lesioned rats

To examine the role of GRK3 in regulating L-DOPA-induced behavior, we employed lentivirus-mediated gene transfer to modulate the level of GRK3 in the DA-depleted striatum. First, we tested whether overexpression of GRK3 affects L-DOPA-induced contralateral rotations in unilaterally 6-hydroxydopamine-lesioned (hemiparkinsonian) rats. We measured the rotation frequency in rats expressing either GFP (control) or GFP-tagged GRK3 in the motor striatum on the lesioned side.

**Figure 1:**
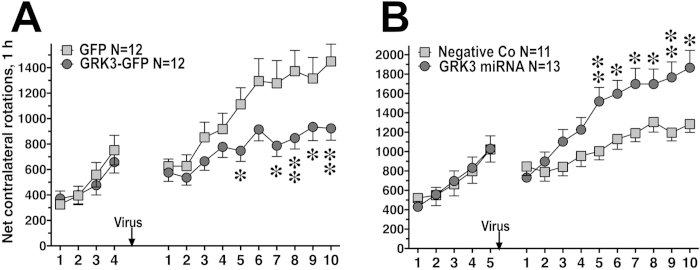
The expression of GRK3 in the lesioned striatum inhibits and knockdown of GRK3 exacerbates rotations in the hemiparkinsonian rat. Data are shown as means ± S.E.M. (**A**) Frequency (for 1 h) of L-DOPA-induced net contralateral rotations (contralateral – ipsilateral) upon repeated L-DOPA treatment in rats expressing GRK3-GFP pre-sensitized with L-DOPA for 5 days before virus injection, as compared to animals expressing GFP (control). Overexpression of GRK3 reduced the frequency of L-DOPA-induced rotations across sessions (p = 0.041) and suppressed the rate of behavioral sensitization to L-DOPA (reduced slope of increase, p < 0.0001). Note similar rotation frequencies and sensitization rate in both groups before the virus injection. (**B**) L-DOPA-induced rotation frequencies in rats injected with the GRK3 miRNA virus as compared to negative control miRNA (GFP). The animals were pre-sensitized with L-DOPA for 5 days before the virus injection. ^*^ - p < 0.05, ^**^ - p < 0.001 between the GRK3 miRNA and control groups on individual days, post hoc Student’s unpaired t-test.

The effect of GRK3 overexpression on frequency of L-DOPA-induced rotations was tested in the behavioral paradigm that involved pretesting the animals for rotations for 5 days before virus injection and then testing them following the injections for 10 more days, as we described previously^25^. The pre-injection rotation frequencies were the same in the control and GRK3 groups, with both groups showing marked behavioral sensitization. However, after the injection rotation frequency in the GRK3 group was reduced across the testing sessions (F(1,198) = 4.72, p = 0.041), and behavioral sensitization was markedly diminished, as evidenced by significant Group x Session interaction (p < 0.0001) (Fig. 1A).

**Figure 2:**
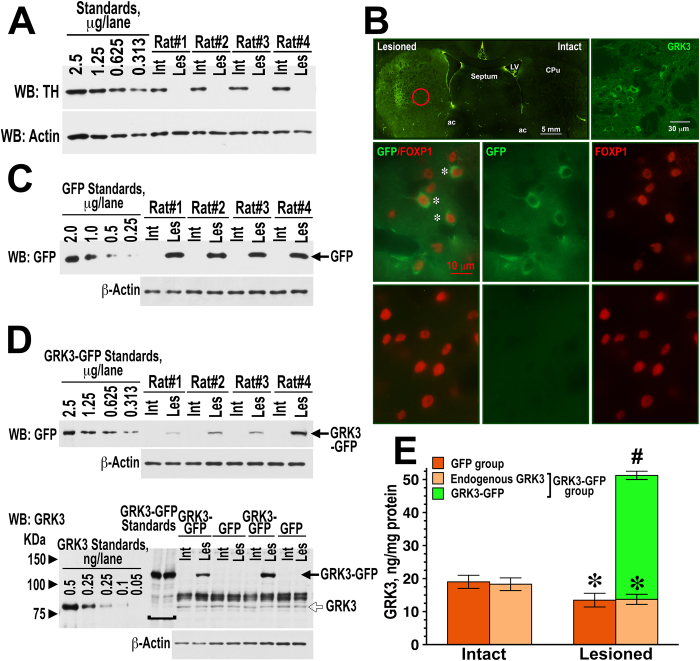
The lentiviral gene transfer induces GRK3 expression in dopamine-depleted rat striatum. (**A**) Loss of dopaminergic innervation in the striatum detected by Western blots for TH in the lesioned (Les) and intact (Int) striata in animals expressing GFP or GRK3. Numbers refer to individual animals. Left 4 lanes show standards obtained by serial dilutions of the striatal tissue lysate. The numbers above indicate the total amount of striatal protein loaded per lane. Actin blot obtained by stripping and re-probing the TH blot shows equal loading. (**B**) Immunohistochemical detection of GRK3-GFP expression. Low power photomicrograph of the rat brain with GRK3-GFP expression detected with anti-GFP antibody (upper panel left). The photomicrograph on the right in the upper panel (at approximate location indicated by the red circle in the lesioned striatum) shows GRK3-GFP-expressing cells at higher magnification. Middle panel shows expression of GRK3-GFP in medium spiny neurons co-labeled for FOXP1 and lower panel – FOXP1-positive neurons in the intact, uninfected striatum. Asterisks indicate double-labeled cells. (**C**) Detection of the GFP expression by Western blot in the lesioned striatum of control rats infected with the GFP lentivirus. The samples from the intact uninfected striata are shown for comparison. Numbers refer to individual animals. (**D**) The expression of GRK3-GFP (upper panel) in four rats in the intact uninfected and lesioned infected striata detected with anti-GFP antibody is shown. Serial dilutions of HEK293 cell lysates infected with GRK3-GFP lentivirus were used as standards. Lower panel shows detection of endogenous GRK3 and GRK3-GFP in GFP- and GRK3-GFP-injected animals with anti-GRK3 antibody. Serial dilutions of purified human GRK3 were used as standards for endogenous GRK3. Lysate of HEK293 cells infected with GRK3-GFP lentivirus was used to indicate the position of GRK3-GFP. (**E**) Quantification of the Western blot data for the expression of GRK3-GFP and endogenous GRK3 in the intact and lesioned hemisphere was performed, as described in Methods. The expression of endogenous GRK3 and transgenic GRK3-GFP in the intact and lesioned (infected) hemisphere was compared by paired Student’s t-test. ^*^ - p < 0.01 to the intact striatum for endogenous GRK3; ^#^ - p < 0.01 to the intact striatum.

Post-mortem examination revealed that both control and GRK3 groups had similarly extensive lesions (Fig. 2A). Less than 2% of the tyrosine-hydroxylase-positive terminals remained in the lesioned caudate-putamen (CPu). The GRK3-GFP lentivirus induced GRK3 expression throughout the dorsolateral CPu (Fig. 2B). GRK3-GFP expression was detected in medium spiny neurons, as determined by double immunohistochemistry for GFP and the marker of these neurons, forkhead box P1 (FOXP1) (Fig. 2B). FOXP1 is a nuclear protein expressed in medium spiny neurons but not in interneurons^29^. Western blots (Fig. 2C) confirmed the presence of expressed GRK3-GFP in the infected striatum. Quantification of GRK3 demonstrated that gene transfer increased the total amount of GRK3 in the lesioned striatum ~4-fold (Fig. 2D).

**Figure 3:**
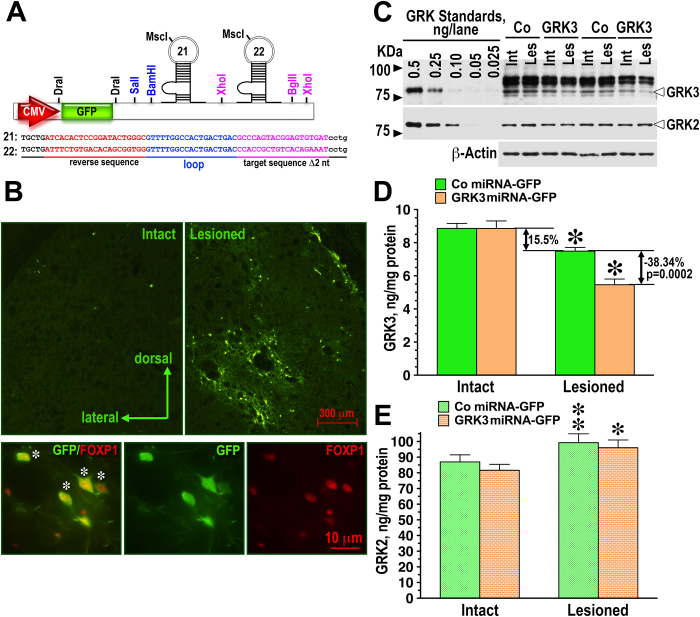
Knockdown of GRK3 in the rat striatum with the miRNA. (**A**) Schematic representation of the lentivirus carrying two chained GRK3 miRNA sequences. (**B**) Representative low magnification photomicrograph of the rat intact control striatum (left upper panel) and the striatum infected with the GRK3 miRNA lentivirus (right upper panel). GFP is expressed co-cistronically with GRK3 miRNA in medium spiny striatal neurons, as demonstrated by double staining for GFP and FOXP1 as described in Methods (lower panels). Middle panel shows the expression of GFP in the lesioned infected striatum, right panel – FOXP1-positive cells; left panel – overlay. Asterisks indicate double-labeled cells. (**C**) Detection of the GRK3 expression by Western blot in the lesioned (Les) hemisphere infected with the GRK3 miRNA (GRK3) or control miRNA (Co) virus in comparison with the intact (uninfected; Int) hemisphere. Five left lanes are different dilutions of purified recombinant human GRK3 used as standards. The arrowhead points to the GRK3 band (the upper bands are nonspecific). (**D**) Quantification of the Western blot data for the expression of GRK3 in the intact and lesioned hemisphere. The expression of endogenous GRK3 and in the intact and lesioned (infected) hemisphere was compared by paired t-test: ^*^ - p < 0.001 to the intact striatum. The expression of endogenous GRK3 in the lesioned (infected) hemispheres of rats infected with the negative control (Co miRNA) and GRK3 miRNA viruses was compared by unpaired t-test (p < 0.05).

To evaluate the role of endogenous GRK3, we tested whether its knockdown with lentivirus-delivered GRK3-selective microRNA (miRNA) influences the behavioral effects of L-DOPA. We used lentivirus carrying two miRNA sequences directed against different regions of GRK3 mRNA and co-cistronic GFP to label infected cells (Fig. 3A). A lentivirus encoding nonsense miRNA and GFP served as control. In the behavioral setting described above, knockdown of GRK3 significantly enhanced the rotation frequency across sessions (F(1,198) = 4.86, p = 0.039) and increased the sensitization to L-DOPA (p < 0.0001) (Fig. 1B). To measure the degree of GRK3 knockdown, we compared the levels of GRK3 in rats expressing GRK3 miRNA and negative control miRNA. Postmortem examination of the infected striatum detected GFP (which was co-cistronically expressed with miRNA) in FOXP1-positive medium spiny striatal neurons (Fig. 3B). The expression of endogenous GRK3 was significantly decreased in the lesioned, as compared to the intact striatum (Fig. 3D), in agreement with our previous report^26^. GRK3 concentration was further significantly decreased by the GRK3 miRNA, as compared to the control lentivirus, by approximately by 40% (Fig. 3B,D). In contrast to GRK3, we detected a small increase in the concentration of GRK2, a closely related GRK isoform, caused by chronic L-DOPA treatment (Fig. 3E), as we reported previously^26^. The concentration of GRK2 was not affected by the GRK3 miRNA. Thus, increased concentration of GRK3 reduced, whereas decreased availability of GRK3 increased L-DOPA-induced rotations and sensitization to L-DOPA.

### Kinase activity is not required for the suppression of L-DOPA-induced rotations by GRK3

**Figure 4:**
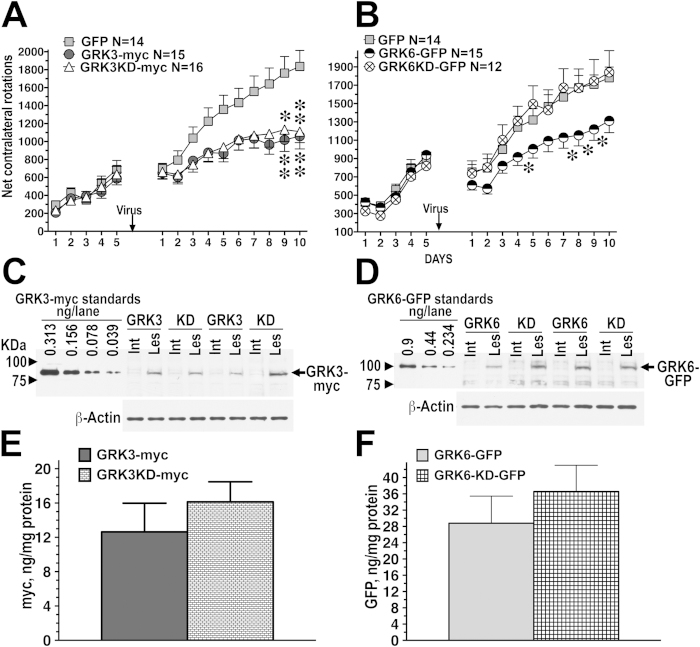
The rotation-suppressing effect of GRK3 is phosphorylation-independent, whereas that of GRK6 requires kinase activity. Data are shown as means ± S.E.M. (**A**) Frequency of L-DOPA-induced net contralateral rotations in rats expressing WT GRK3-myc or GRK3-K220R kinase-dead mutant (GRK3KD-myc), as compared to animals expressing GFP (control). Both WT GRK3 and GRK3KD significantly reduced overall rotation frequency (p < 0.05) and the slope of behavioral sensitization (p < 0.001). (**B**) Frequency of L-DOPA-induced net contralateral rotations in rats expressing GRK6 or GRK6-K215,216M kinase-dead mutant (GRK6KD-GFP), as compared to animals expressing GFP (control). ^*^ - p < 0.05, ^**^ - p < 0.01 to the GFP group, Bonferroni/Dunn post-hoc test for individual days. (**C**) Expression of GRK3 and GRK3KD in the infected striatum detected with anti-myc antibody. The graph shows quantification of the Western blot data (**D**) Expression of GRK6 and GRK6KD detected by anti GFP antibody. (**E**) and (**F**) quantification of the Western blot data on GRK3 and GRK6 expression, respectively.

GRKs phosphorylate active GPCRs, thereby promoting the binding of arrestins, inducing receptor desensitization [reviewed in^8^] and facilitating arrestin-mediated signaling^4^,^5^. In order to determine whether kinase activity of GRK3 is required for its anti-rotation effect, we constructed lentivirus encoding kinase-dead (KD) GRK3-K220R mutant^30^ tagged with myc epitope for easy detection. The myc-tagged WT GRK3 was fully functional and the mutant possessed no detectable catalytic activity, as determined by in cell phosphorylation assay using the dopamine D1 receptor (D1R) as a substrate (Fig. S1). We compared the effect of the overexpression of WT and kinase-dead GRK3 on L-DOPA-induced rotations. To our surprise, we found that kinase-dead GRK3-K220R was just as effective in suppressing rotations as WT GRK3 (Fig. 4A). Two-way repeated measure ANOVA, with Group (GRK3, GRK3-KD, or GFP) as between group factor and Session (as within group factor) yielded significant effect of group [F(2,378) = 4.47, p = 0.017) and Group x Session interaction [F(9,378) = 6.56, p < 0.0001]. Post hoc comparison (Bonferroni’s test with correction for multiple comparisons) revealed that both WT GRK3 and GRK3 KD groups had significantly reduced overall rotation frequencies, as compared to the GFP group (p < 0.05).

We have previously demonstrated that lentivirus-mediated overexpression of another GRK isoform, GRK6, lead to a significant reduction in the rotation frequency and dyskinetic behavior in the rodent and monkey model of LID^25^. Therefore, we examined the contribution of the kinase activity to the effect of GRK6. Preliminary testing demonstrated that the KD GRK6 mutant^30^ GRK6A-K215,216M was devoid of kinase activity at the D1 dopamine receptor (Fig. S1B). We found that, in contrast to GRK3, the kinase activity was indispensible for the anti-rotation effect of GRK6. As shown in Fig. 4B, the KD mutant GRK6-K215,216M was completely ineffective. Two-way repeated measure ANOVA with Group (GRK6, GRK6-RD, or GFP) and Session (as within group factor) yielded significant effect of Group [F(2,360) = 3.66, p = 0.0346) and Group x Session interaction [F(9,360) = 2.24, p = 0.00258] due to the rotation-suppressing effect of WT GRK6. As revealed by post hoc comparison, WT GRK6 group had significantly lower rotation frequencies across sessions than either GFP or GRK6 KD group (p < 0.05). Postmortem determination of the levels of WT and kinase-dead GRK3 and GRK6 demonstrated comparable expression of both constructs of each kinase (Fig. 4C,D).

### The behavioral activity of GRK3 is mediated by the function of its RGS homology domain

**Figure 5:**
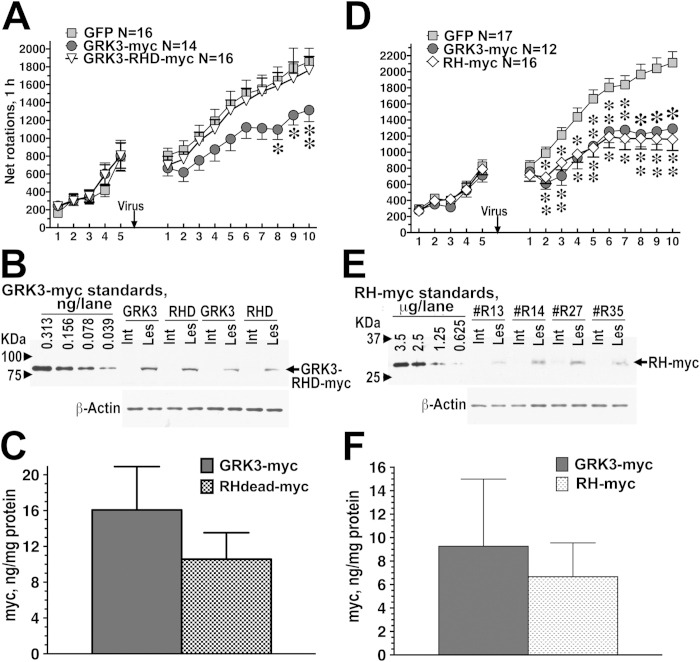
The rotation-suppressing effect of GRK3 is mediated by its RH domain. Data are shown as means ± S.E.M. (**A**) Frequency of L-DOPA-induced rotations in rats expressing WT GRK3-myc or GRK3-R106A,D110A mutant (GRK3-RHD-myc) as compared to GFP (control). ^*^ - p < 0.05 to the GFP group, Bonferroni/Dunn post hoc-test. (**B**) Expression and quantification of GRK3-myc and GRK3-RHD-myc with anti-myc antibody. The panel shows representative Western blot with the myc detection in the intact (Int) and lesioned (Les) striata of two rats expressing GRK3-myc (GRK3) and GRK3-RHD-myc (RHD). Left four lanes – GRK3-myc standards produced by infecting HEK293 cells with GRK3-myc lentivirus and preparing serial dilutions of the cell lysate. The numbers show the amount of cell protein per lane. (**C**) The graph shows quantification of the expression data as mean ± S.E.M. in ng of GRK3 per mg of total protein. (**D**) Frequency of L-DOPA-induced net contralateral rotations in rats expressing WT GRK3 or isolated RH domain (RH-myc), as compared to animals expressing GFP (control). ^*^ - p < 0.05 to the GFP group, Bonferroni/Dunn post hoc test. (**E**) The panel shows representative Western blot with the myc detection in the intact (Int) and lesioned (Les) striata of four rats expressing RH-myc. Left four lanes – RH-myc standards produced by infecting HEK293 cells with RH-myc lentivirus and preparing serial dilutions of the cell lysate. The numbers show the amount of cell protein per lane. (**F**) The graph shows quantification of the expression data as mean ± S.E.M. in equivalents of ng of GRK3-myc per mg of total protein.

In contrast to GRK6, GRK3 possesses two additional functional domains that can interfere with the signaling in a phosphorylation-independent manner (reviewed in^8^). One is functional RGS homology (RH) domain capable of binding and sequestering active Gαq/11, thereby reducing signaling mediated by these G proteins^12^,^13^,^14^,^15^. The second is pleckstrin homology (PH) domain that binds Gβγ and reduces Gβγ-functions^31^,^32^. In order to determine, which function is responsible for anti-rotation effect of GRK3, we used GRK3-R106A,D110A mutant with severely reduced binding to Gαq/11^13^. We found that, in contrast to kinase-dead mutant that retained full activity, the RH-dead GRK3-R106A,D110A mutant (GRK3-RHD) was completely ineffective in suppressing L-DOPA-induced rotations (Fig. 5A) when expressed at the level comparable to that of WT GRK3 (Fig. 5B,C). Two-way repeated measure ANOVA, with Group (GFP, GRK3-WT, GRK3-RHD) as between group factor and Session (as within group factor) yielded significant effect of group [F(2,387) = 4.03, p = 0.0486) and Group x Session interaction [F(9,387) = 2.532, p = 0.0006], both effects due to the rotation-reducing effect of WT GRK3. These data suggest that the effect of GRK3 on L-DOPA-induced rotations was mediated via the function of RH without contribution from PH domain, since PH domain remained intact in the GRK3-RHD mutant.

In order to verify this conclusion, we constructed a lentivirus expressing isolated C-terminally myc-tagged RH domain (N-terminal amino acids 1-175; RH) (Fig S2A). Figure S2B shows the expression of RH(1-175) in the lesioned striatum upon lentivirus infection. Since RH lacked not only the kinase domain but also PH domain required for recruitment of GRK3 to activated GPCRs via interaction with Gβγ^33^, we examined whether it retains proper sub-cellular localization in the brain. We found that overall subcellular localization of RH was similar to that of the parental GRK3 and that it was present at the plasma membrane (Fig. S2C). RH effectively reduced the frequency of L-DOPA-induced rotations (Fig. 5D). Two-way repeated measure ANOVA, with Group (GFP, GRK3 WT, RH) as between group factor and Session (as within group factor) yielded significant effect of group [F(2,378) = 9.4, p = 0.0004) and Group x Session interaction [F(9,378) = 107.6, p < 0.0001], the latter due to a significant reduction in the sensitization slope. The expression level of RH was comparable to that of GRK3 (Fig. 5E,F). The data demonstrate that the anti-rotational effect of GRK3 was mediated via RH domain and ruled out the contribution of other functional domains.

**Figure 6:**
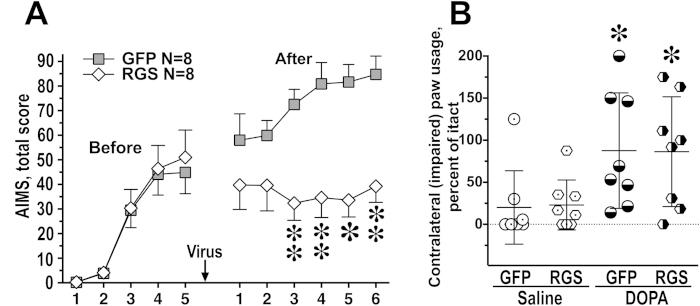
The expression of the RH of GRK3 in the lesioned striatum inhibits abnormal involuntary movements (AIMs) without compromising anti-akinetic effect of L-DOPA. Data are shown as means ± S.E.M. (**A**) Hemiparkinsonian rats were tested for L-DOPA-induced AIMs as described in Methods. The graph shows combined AIMs scores per session in rats expressing GFP or RH treated with L-DOPA before and after the virus injection. ^**^ p < 0.01, ^*^ (large)- p < 0.001 between the GFP and RH groups, Mann-Whitney test. (**B**) The rats were tested in the cylinder test on Days 12 and 13 (when no AIMs testing was conducted), as described in Methods. The scatterplot shows the usage of the paw contralateral to the lesion (injured) as the percentage of the usage of the intact paw. L-DOPA administration (7.5 mg/kg) significantly improved the use of the injured paw equally in the GFP and RGS groups. ^*^ - p < 0.05 to respective saline values by Wilcoxon Signed Rank test.

Next, we tested the effect of the RH overexpression in the motor striatum on the development of abnormal involuntary movements (AIMs). The rats were pretested for AIMs as described previously^25^ and then received the injections of the control (GFP) or RH lentivirus. The expression of RH in the lesioned striatum significantly reduced the AIMs scores in testing sessions 3 through 6 (Fig. 6A). The expression of RH suppressed sensitization to L-DOPA, since the AIMs scores remained essentially the same in the RH groups in the sessions 3 through 6, whereas in the GFP control they significantly increased. We used the cylinder test to examine the effect of RH expression on the anti-akinetic action of DOPA. We found that administration of L-DOPA reduced asymmetry in the use of the contralateral (injured) paw equally in both GFP and RH groups, demonstrating that RH did not compromise therapeutic effect of L-DOPA (Fig. 6B).

### The RGS homology domain of GRK3 altered downstream signaling in the striatum

**Figure 7:**
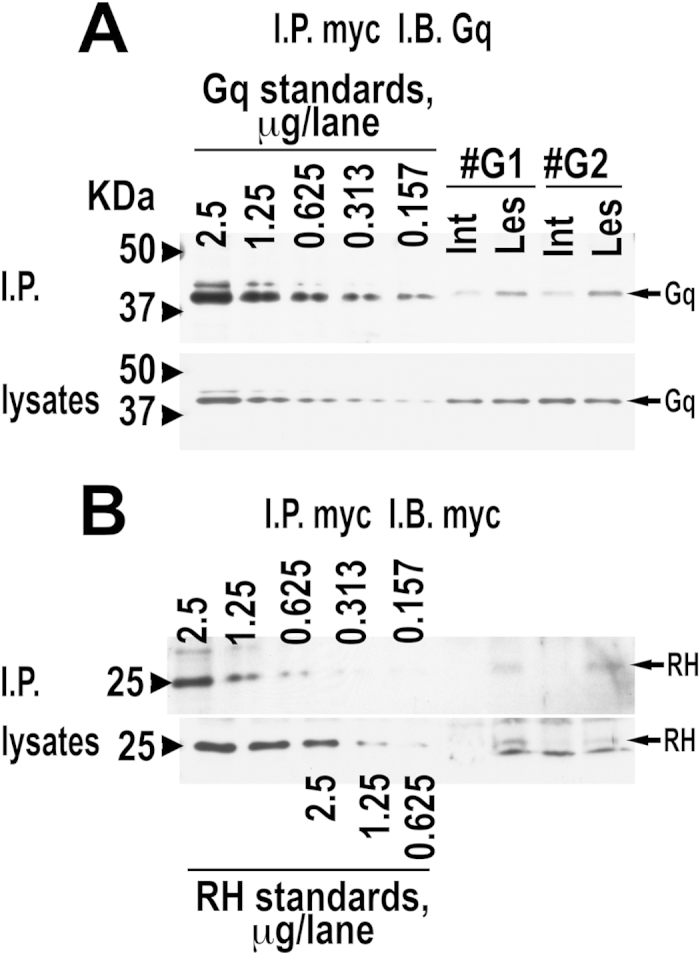
Isolated RH of GRK3 interacts with Gαq in the brain. Co-immunoprecipitation experiments from the rat striata were performed as described in Methods. (**A**) Upper panels show the amount of Gq co-immunoprecipitated with myc in the intact (Int), which was not infected with RH-myc virus and, thus, lacked the bait, and lesioned (Les) striata of two rats. The panel below shows the concentration of Gq in the lysates. Left five lanes – serial dilutions of the HEK293 cell lysate used as standards for Gq. (**B**) Upper panels show the amount of RH-myc immunoprecipitated by anti-myc antibody in the intact (Int), which was not infected with RH-myc virus and, thus, lacked RH-myc, and lesioned (Les) striata of two rats. The panel below shows the concentration of RH in the corresponding lysates of the intact and lesioned striata. Left five lanes - serial dilutions of the lysate of HEK293 cells infected with RH-myc lentivirus used as standards for RH-myc.

To test whether RH suppresses L-DOPA-induced rotations via interaction with Gαq, we used immunoprecipitation to detect possible interaction of lentivirally delivered RH with endogenous Gαq in the brain. We found that endogenous Gαq co-immunoprecipitated with myc-RH from the lesioned striatum (Fig. 7).

**Figure 8:**
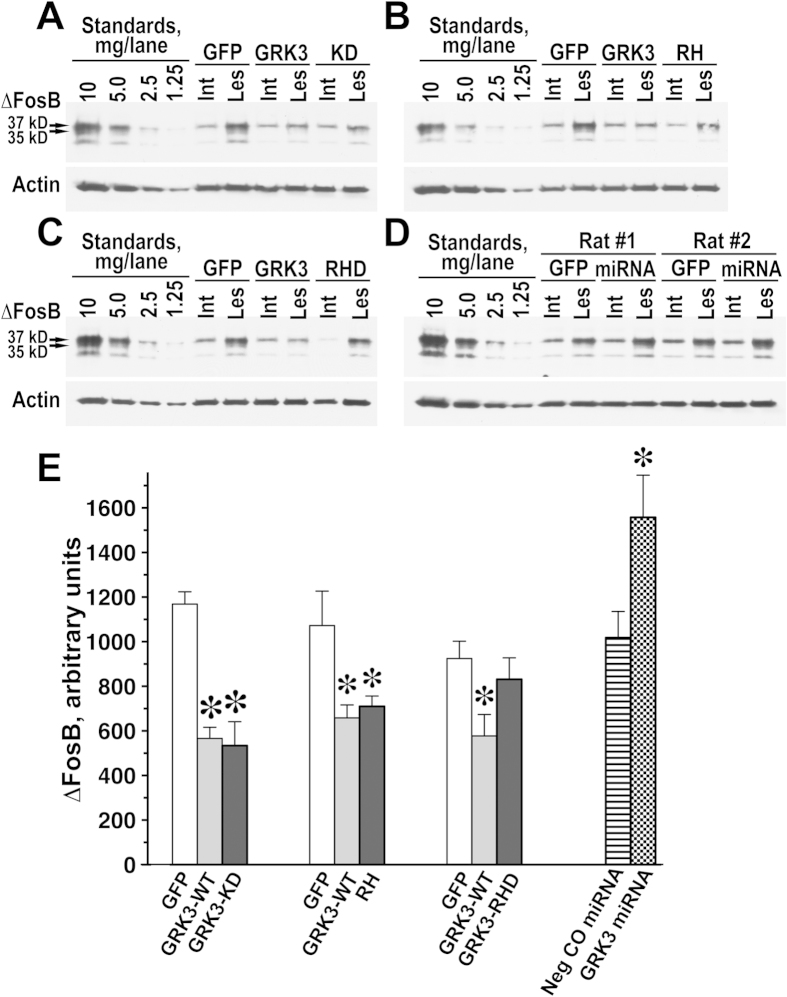
GRK3 acting via its RH domain reduces accumulation of ∆FosB in the lesioned striatum in rats chronically treated with L-DOPA. (**A**–**D**) Representative Western blot showing relative accumulation of ∆FosB in different experimental groups in the intact (Int) and lesioned (Les) striata. The experiments were performed as described in Methods. (**A**) Comparison of the effect on the ∆FosB level of the striatal overexpression of WT GRK3 with that of kinase-dead GRK3-K220R mutant (KD). (**B**) Comparative effect on the ∆FosB level of WT GRKs and isolated RH domain of GRK3 (RH). (**C**) The effect of the striatal expression of WT GRKs (GRK3) and GRK3-R106A,D110A mutant with the function of RH domain disabled (RHD) on the ∆FosB accumulation. (**D**) Modulation of the ∆FosB level by striatal knockdown of GRK3 via expression of GRK3 miRNA as compared to the negative control nonsense miRNA (Neg CO miRNA). (**E**) Quantification of the level of ∆FosB in the lesioned striatum in rats expressing GFP, WT GRK3 (GRK3-WT), kinase-dead GRK3 (GRK3-KD), isolated RH of GRK3 (RH), or RH-dead GRK3 mutant (GRK3-RHD). Additionally, quantification of ∆FosB in rats with knockdown of GRK3 via GRK3 miRNA, as compared to the negative control miRNA (Neg CO miRNA) is shown. ^*^ (large) - p < 0.01, ^*^ (small) - p < 0.05 to respective controls.

Chronic L-DOPA treatment causes the accumulation of the transcription factor ∆FosB in the lesioned striatum, the process that parallels the development of behavioral sensitization to L-DOPA and LID^34^. Therefore, we examined the effects of the GRK3 constructs on the ∆FosB accumulation. Overexpression of WT GRK3 (GRK3-WT) and kinase-dead GRK3 (GRK3-KD) significantly and to a similar degree reduced accumulation of ∆FosB in the lesioned striatum, as compared to the GFP-expressing control (GFP-CO) (Fig. 8A,E), the effect that paralleled the behavioral changes caused by these proteins. The isolated RH of GRK3 (RH) also reduced the ∆FosB accumulation similarly to WT GRK3, whereas GRK3-RHD was ineffective (Fig. 8B,C,E). Conversely, knockdown of GRK3 via GRK3 miRNA increased the level of ∆FosB in the lesioned striatum as compared to the negative control miRNA (Neg CO miRNA) (Fig. 8D,E). Thus, the effects of the level of GRK3 and its RH domain on ∆FosB clearly parallel their effects on behavioral manifestations of LID.

## Discussion

Loss of DA in PD and in animal models of PD causes motor defects as well as multiple changes in signaling in striatal neurons. Following DA depletion, many signaling pathways acquire enhanced sensitivity to dopaminergic stimulation^16^,^17^,^24^. The supersensitivity of the D1 receptors signaling is a conspicuous result of the loss of DA^17^,^20^. Chronic treatment with L-DOPA aimed at restoring missing DA to the striatum normalizes some of the signaling pathways but further deregulates, or fails to normalize, others (16,20,24,25, see also^35^ and references therein), which eventually results in dyskinesia and other motor complications. Exaggerated signaling of the striatal D1 DA receptors appears to be the main contributor to LID^20^,^21^, although enhanced activity of D2^22^, and D3^25^,^36^ receptors has also been implicated in LID in rodents and primates. These data strongly suggest that normalization of this excessive signaling may provide anti-LID benefits. However, since dopaminergic signaling is required for proper movement control, the challenge is to suppress signaling responsible for LID while preserving enough dopaminergic activity to support the antiparkinsonian action of the drug. One mechanism that could accomplish that task is GRK-arrestin-dependent homologous desensitization of GPCRs. Due to selectivity of GRKs for activated GPCRs^8^, receptors are able to activate G proteins before the signaling is shut down by GRK-dependent phosphorylation followed by high affinity arrestin binding.

We hypothesized that DA depletion brings about a deficit in the desensitization mechanism, likely as a compensatory measure to sustain the declining signaling. When L-DOPA is applied, remaining desensitization machinery is inadequate to cope with the signaling load, resulting in exaggerated activity of the striatal signaling pathways that contributes to LID. In support of that hypothesis, we found reduced concentration of GRK6 and GRK3 in the DA-depleted striatum in the 6-OHDA-lesioned rats; that reduction persisted through the chronic L-DOPA treatment^26^. Furthermore, we demonstrated that promoting GPCR desensitization in the DA-depleted striatum via virus-mediated overexpression of GRK6 ameliorated LID in both primate and rodent models without compromising the antiparkinsonian effects of L-DOPA^25^ and normalized the activity of striatal signaling pathways rendered abnormal by loss of dopamine^19^,^25^. The reduced availability of GRK6 in the lesioned striatum appears to significantly contribute to both hyperactive signaling and LID, since knockdown of GRK6 in the DA-depleted striatum aggravated LID^25^. These data are in agreement with the finding that GRK-dependent phosphorylation and not arrestin binding is the rate-limiting step in GPCR desensitization^37^. Our data are in line with earlier reports showing that modulation of the GRK availability in cultured cells and *in vivo* has a profound effect on the GPCR signaling^38^,^39^,^40^,^41^,^42^.

Here we examined the function of another GRK isoform, GRK3, out of four expressed by striatal neurons^26^,^27^, that is consistently downregulated by the loss of DA^19^,^25^,^26^. We found that, similarly to GRK6, lentiviral overexpression of GRK3 in the lesioned striatum suppressed and knockdown enhanced L-DOPA-induced rotational behavior in hemiparkinsonian rats (Fig. 1). If the action of GRK3 is mediated by receptor phosphorylation, it could be due to facilitated receptor desensitization or enhanced arrestin-mediated signaling upon arrestin binding to phosphorylated receptors^4^,^5^. However, in contrast to GRK6 that acted via receptor phosphorylation, the action of GRK3 was phosphorylation-independent, mediated by its RH domain (Figs. 4,5), which effectively ruled out both phosphorylation-dependent desensitization and arrestin-dependent signaling. The kinase domain of all GRKs is inserted in a loop within the RH domain structure, which is a very unusual feature for multi-domain proteins^8^,^43^. The RH domain of GRKs was originally identified *in silico*^6^ but later demonstrated to be functionally active in cultured cells^9^,^10^,^11^,^12^,^13^,^44^. In contrast to “conventional” RGS proteins, many of which accelerate GTP hydrolysis by G proteins^7^, the RH of GRKs possess almost no GTPase accelerating activity but binds and sequesters active Gαq/11, thereby reducing the signaling via Gq/11-coupled GPCRs^9^,^10^,^12^,^13^. The fact that GRK6 was proven to act exclusively via phosphorylation is not surprising. Although all GRKs possess the RH domain, only the RH domains of GRK2 and GRK3 seem to be capable of binding Gαq/11^9^. The structure of GRK6 revealed that its RH domain has a shorter α5 helix than that of GRKs2/3^43^ and lacks structural elements known to be required for binding to Gαq/11^11^,^12^,^13^.

The experiments presented here provide definitive proof that the functional RH domain is necessary and sufficient for the anti-LID activity of GRK3. The GRK3 construct with inactivated RH domain but with other domains functionally intact (GRK3-RHD) did not affect L-DOPA-induced rotations, demonstrating that functional RH domain is required for the GRK3 anti-LID activity, whereas other domains do not make a measurable contribution. Furthermore, isolated RH domain lacking other domains recapitulated the effect of full-length GRK3, further supporting the conclusion that RH domain is sufficient for the GRK3 action. Moreover, in the context of similar subcellular localization of full-length GRK3 and its separated RH domain, the finding that both show the same anti-LID activity strongly suggests that full-length GRK3 exerts its effect in this paradigm via its RH domain. Interestingly, the effect of GRK3 on the accumulation of ∆FosB, the transcription factor implicated in LID development^34^,^45^, is phosphorylation-independent and is mediated via its RH domain function (Fig. 8). Since the RH of GRK3 is only capable of interacting with Gαq/11^9^, these data for the first time implicate Gq-mediated signaling in the L-DOPA-induced ∆FosB accumulation. Gq-mediated signaling could affect ∆FosB accumulation via modulation of the protein kinase C^46^ or calcium/calmodulin-dependent protein kinase II (CaMKII) activity^47^.

GRK6 likely modulated DA-dependent behavior by facilitating desensitization of DA receptors. Previous work with GRK6 knockout mice has demonstrated that GRK6 is the key isoform regulating the signaling via the DA receptors^39^. Our results supported that conclusion although emphasizing the role of the D1 receptor regulation by GRK6 in addition to that of the D2 receptor^25^. With GRK3, the situation is different. Neither D1R nor D2R were traditionally thought to couple to Gq/11. However, recent data demonstrated that D1R expressed on direct pathway medium spiny neurons^48^,^49^ and D5R expressed on cholinergic interneurons^50^,^51^ can couple to Gq^52^,^53^. Additionally, D1R/D2R dimers have been reported to couple to Gq^54^,^55^. However, dimer formation requires D1R/D2R co-expression, and only a limited proportion (5–7%) of MSN in the dorsal striatum co-express both receptors^56^,^57^,^58^. Since RH directly binds active Gαq/11, its action does not depend on GRK3 binding to a particular receptor. GRK3 could sequester via it RH active Gαq generated by activation of a number of non-DA Gq/11-coupled receptors expressed in striatal neurons. For example, active Gαq/11 could be generated by Group I mGluRs indirectly activated by L-DOPA. mGluR1 and, particularly, mGluR5 is highly expressed in medium spiny striatal neurons and interneurons^59^,^60^,^61^,^62^. Studies showing that drugs targeting these receptors modulate L-DOPA-induced behaviors and LID^63^,^64^ support the involvement of these receptors in the action of L-DOPA. Other Gq-coupled receptors could also make a contribution, such as 5-HT2A receptors expressed by medium spiny striatal neurons^65^. Antagonists of these receptors reduce LID, suggesting a role in L-DOPA action, although it is unclear whether the mechanism is pre- or postsynaptic^66^. A major Gq-coupled receptor expressed by both types of medium spiny striatal neurons, but not by interneurons, is the M1 muscarinic receptor^67^,^68^. Anticholinergic drugs targeting this receptor are used to treat stiffness and tremor in PD^68^, but so far there is no evidence of its involvement in LID.

The results of these experiments demonstrate that the mechanisms responsible for the desensitization of the GPCR signaling are critically involved in the development of LID and can be targeted for anti-LID therapy. An enhancement of the capacity of the GPCR desensitization machinery both at the level of receptor-G protein interaction and G protein inactivation can reduce DA depletion-induced receptor supersensitivity and relieve LID. Our data identify GRK3 for the first time as a therapeutic target in LID. Importantly, RH domain overexpressed in the lesioned motor striatum alleviated LID without compromising therapeutic effect of L-DOPA, as measured by the cylinder test. Furthermore, the results suggest that GPCR desensitization machinery and, specifically, GRK isoforms could be targeted for judicial manipulation of the receptor signaling in numerous disorders associated with aberrant signaling via G protein-coupled receptors, from schizophrenia to drug abuse. Distinct mechanisms involved in GRK6 and GRK3 actions suggest that simultaneously targeting these two GRKs is likely to provide additive benefits.

## Methods

### Virus construction and preparation

The full-length coding sequence of the rat GRK3 (gi 6978467) C-terminally tagged with GFP or GFP alone (control) was cloned into the lentiviral vector pLenti6/V5-DEST, and the virus was produced using ViraPower system (Invitrogen, Carlsbad, CA), as described previously^25^. The lentivirus encoding full-length rat GRK6C (gi 163310714, equivalent of human GRK6A), described previously, was used^25^. Kinase-dead form of GRK6, GRK6A-K215, 216M^30^, kinase-dead GRK3, GRK3-K220R^30^, GRK3 with inactive RH domain GRK3-R106A,D110A^11^, and isolated RH(1-175)^11^ were produced by mutagenesis, and mutations were verified by sequencing. Four validated microRNAs targeting rat GRK3 were purchased from Invitrogen (Carlsbad, CA) and pretested in cultured HEK293 cells expressing rat GRK3. Two most active miRNAs were chained in the lentiviral clone, as described^25^, and miRNA-encoding virus was produced with Block-iT HiPerform Lentiviral Pol II RNAi expression system (Invitrogen). Viral titers were measured based on GFP or myc expression using HEK293 cells infected with appropriated lentiviruses.

### Rat surgeries and virus injection

Adult Sprague-Dawley rats (Charles River) were used. The animals were housed at the Vanderbilt University animal facility in a 12/12 light/dark cycle with free access to food and water. All procedures were performed in accordance with the NIH Guide for Care and Use of Laboratory Animals. The procedures were approved by the Vanderbilt University IACUC. The 6-OHDA lesion was performed, as described^16^,^26^. Briefly, rats were deeply anesthetized with ketamine/xylasine (60/6 mg/kg i.p.) and mounted on a stereotaxis. Rats were treated with desimipramine (25 mg/kg i.p.) 30 min. prior to infusion of 6-hydroxydopamine (6-OHDA). 6-OHDA (8 mg in 4 ml of 0.05% ascorbic acid) was infused unilaterally into the medial forebrain bundle at coordinates A = −4.3 mm, L = 1.2 mm, H = −8.5 mm. The viruses were injected after the behavioral pre-testing. The rats were anesthetized with ketamine/xylazine (60/6 mg/kg i.p.) and mounted on a stereotaxis. The virus injection (5 μl of the concentrated virus in saline per striatum 0.3 μl/min) was made unilaterally (on the 6-OHDA-lesioned side) into the dorso-lateral caudate-putamen at coordinates AP 0.2; ML 3.5; DV 5.7.

### Rat behavior

#### Rotations

Three weeks after the 6-OHDA lesion, the animals were tested for rotational response to apomorphine (0.05 mg/kg s.c.) for 1 h using an automated rotometer (AccuScan Instruments). Both ipsilateral and contralateral 360° turns were recorded, and the net rotational asymmetry (contralateral minus ipsilateral turns) was calculated. The animals were then treated with L-DOPA (L-3,4-Dyhydroxyphenylalanine methyl ester hydrochloride, 25 mg/kg i.p. twice daily, morning and afternoon) for 5 days, and the rotational response was recorded every day after the morning injection (pre-testing). Five days after the virus injections, the rats were again treated with L-DOPA (25 mg/kg i.p. twice daily) and tested for L-DOPA-induced rotations every day for 10 days, as described previously^25^.

#### Abnormal Involuntary Movements (AIMs)

Testing for AIMs was performed essentially as described previously^25^. Briefly, rats were treated with increasing doses of L-DOPA (2–10 mg/kg day) for 15 days, starting 3 weeks after the 6-OHDA lesion. The dose regiment was adjusted for each individual animal until substantial AIMs scores were achieved. The virus injection was performed on day 14, and the rats were allowed to recover for 5 days before resuming the treatment for 18 more days with 10 mg/kg L-DOPA. AIMs were assessed by independent observer in a blind manner on 0–4 scale^69^ on every third day: 0 – no AIMs; 1 – infrequent AIMs occurring <50% of the time; 2 – frequent AIMs occurring > 50% of the time; 3 – constantly present AIMs that are interrupted by external stimulation (tap on the cage); 4 – constant AIMs that are insensitive to external stimulation. AIMs of four categories were scored (orolingual, limb, locomotor, and axial) for 1 min every 20 min for 3 h (total 9 observations; maximal score for each observation 16, maximal total score per session 144). The cylinder test was performed on Days 8 and 9 between AIMs sessions 3 and 4. Randomly selected half of the GFP and RH rats were injected with saline or with L-DOPA (7.5 mg/kg). The next day, the order was reversed. Rats were places in a plexyglass cylinder (30 cm in diameter), and their behavior recorded on video camera under red light for 6 min. The number of times the rat used the contralateral (injured) or ipsilateral paw for support were counted (use is defined as the animal supporting its body with digits extended) from the recordings by an observer blind to the animal’s treatment. Preferential use of the paw ipsilateral to the lesion (controlled by the intact hemisphere) is indicative of the “parkinsonian” defect. L-DOPA administration enhances the use of the contralateral paw, controlled by the “lesioned” hemisphere, measuring antiparkinsonian effect of the drug^63^,^70^,^71^. The results are expressed as percentage of the contralateral paw usage relative to the ipsilateral paw.

### Tissue preparation

Upon completion of the drug administration and behavioral testing, the rats were decapitated under isoflurane anesthesia, and their brains were collected and rapidly frozen on dry ice. The midbrain containing the substantia nigra was dissected and post-fixed in 4% paraformaldehyde for TH immunohistochemistry to determine the loss of dopaminergic neurons. The rostral parts of the brains containing the striata were kept at −80 °C until samples were collected for Western blot analysis. To collect the samples for Western blot, the brains were cut through the striatum, the position of the virus injection track was identified, and the tissue around the injection track was collected to determine the expression of virus-encoded proteins and TH concentration as described previously^19^,^25^. Randomly selected animals were overdosed with ketamine/xylazine and transcardially perfused with 4% paraformaldehyde. The brains were removed, post-fixed in 4% paraformaldehyde, cryoprotected, and frozen at −80 °C until used for detection of infected cells by immunohistochemistry.

### Immunohistochemistry and Western blot

The lesion quality was determined by TH immunohistochemistry and Western blot for TH. The midbrain sections containing the substantia nigra were stained with rabbit anti-TH antibody (Chemicon, Temecula, CA) (1:1000 overnight at 4 °C) followed anti-rabbit secondary antibody conjugated with Alexa Fluor 488. Western blot for GRK3-GFP was performed with mouse anti-GFP antibody (Clontech, Mountain View, CA) at dilution 1:2,000. To quantify GRK3 overexpression in comparison with the endogenous level, the GRK3 concentration was measured by Western blot using rabbit polyclonal anti-GRK3 antibody directed against the C-terminus of human GRK3 from Santa Cruz Biotechnology (Santa Cruz, CA) (1:500). The expression of myc-tagged GRK3 constructs was measured with rabbit anti-myc (Cell Signaling Technology, Beverly, MA) at 1:1,000 dilution.

For immunohistochemistry, mouse anti-GFP (Clontech) primary antibody was used. The sections were incubated with the primary antibody (1:500 dilution) overnight at 4 °C followed by goat anti-rabbit biotinylated secondary antibody and streptavidin conjugated with Alexa Fluor 488 (Invitrogen, Carlsbad, CA). For double labeling with the marker of medium spiny neurons forkhead box P1 (FOXP1)^29^, the sections were labeled with mouse anti-GFP (1:500) antibody and rabbit anti-FOXP1 antibody (Cell Signaling Technology) (1:400 dilution) and detected by biotinylated secondary antibody/streptavidin-Alexa Fluor 488 (GFP; green) and anti-rabbit-Alexa Fluor 568 (FOXP1; red). The sections were photographed at low magnification on Nikon TE2000-E automated microscope with 4x dry objective and Q-Imaging color digital camera using stitching function of Nikon NIS-Elements software. High power photographs were collected on LSM710 confocal microscope in green and red channels with 40x oil immersion objective at 576 × 576 pixels. The images were assembled in Photoshop CS6, with minimal contrast adjustments applied separately to channels to equalize their intensity.

### Immunoprecipitation from the striatal tissue

The 6-OHDA-lesioned rats were injected into the lesioned striata with lentivirus encoding myc-tagged RH. The intact striata were not infected and served as the no-bait control. Both lesioned and intact striata (the lesioned striata were collected around the injection tract and the intact – at matched level) from each rat were dissected and lysed in the NP-40-containing immunoprecipitation (IP) buffer, as described previously^72^. The lysates were cleared by centrifugation, adjusted by protein content with IP buffer, and supernatant was incubated overnight at 4 °C with mouse anti-myc antibody conjugated to agarose beads (Sigma-Aldrich, St. Louis, MO). The resin was washed 3 times with IP buffer in minicolumns, and samples were eluted with SDS buffer. Samples were analyzed by Western blot with rabbit anti-Gq/11 and rabbit anti-myc antibodies.

### In cell receptor phosphorylation

D1 dopamine (D1R) (expression constructs were described previously^73^) was co-expressed with full-length WT or mutant GRK3 or GRK6 (kindly provided by Dr. J. L. Benovic, Thomas Jefferson University) in HEK293-FT cells. The mutations to render GRK3 and GRK6 kinase-dead (GRK3-K220R and GRK6-K215,216M) were introduced by PCR and confirmed by dideoxy-sequencing. In-cell receptor phosphorylation experiments was performed as described previously^74^. Details of the methods are described in Supplemental Information.

### Fractionation

Rats were injected with lentiviruses encoding either WT GRK3-myc or myc-RH. The striatal tissue was isolated as fractioned as described previously^25^,^26^. Details of the methods are described in Supplemental Information.

### Data Analysis

The rotation data were analyzed by two-way repeated measure ANOVA with Group (GFP versus GRK3 and GRK3 mutants, or GRK6 and GRK6-KD) as a between group and Session as a repeated measure factor. The group differences across sessions were assessed by post hoc Bonferroni/Dunn test with correction for multiple comparisons. When significant effect of Group was observed, the data for individual sessions were compared by unpaired Student’s test (two groups) or by Bonferroni/Dunn post hoc test. The AIMs scores for each session were compared by Mann-Whitney nonparametric test. The data for the cylinder test were analyzed using nonparametric paired Wilcoxon Signed Rank test (to evaluate the effect of L-DOPA) or Mann-Whitney (for group comparison). The value of p < 0.05 was considered significant.

## Additional Information

**How to cite this article**: Ahmed, M. R. *et al*. GRK3 suppresses L-DOPA-induced dyskinesia in the rat model of Parkinson's disease via its RGS homology domain. *Sci. Rep*. **5**, 10920; doi: 10.1038/srep10920 (2015).

## Supplementary Material

Supplementary Information
