# Supplementary material for: GRK3 suppresses L-DOPA-induced dyskinesia in the rat model of Parkinson’s disease via its RGS homology domain
Source: Sci Rep. 2015 Jun 4;5:10920. doi: 10.1038/srep10920 (PMC4455246; doi:10.1038/srep10920)
Supplement: Supplementary Information [file srep10920-s1.pdf]

GRK3 suppresses L-DOPA-induced dyskinesia in the rat model of Parkinson's disease via its RGS homology domain

Mohamed R. Ahmed, Evgeny Bychkov[1]*, Lingyong Li*[2]*, Vsevolod V. Gurevich and Eugenia V. Gurevich*[*]
Department of Pharmacology, Vanderbilt University, Nashville, TN 37232;

1 - Current address: Department of Pharmacology, Military Medical Academy, Saint-Petersburg, Russia 194175;

2 - Current address: Department of Anesthesiology and Perioperative Medicine, University of Texas MD Anderson Cancer Center, Houston, TX

* Corresponding author
Eugenia V Gurevich, Department of Pharmacology, Vanderbilt University, 2200 Pierce Ave., PRB422, Nashville, TN 37232; e-mail: Eugenia.Gurevich@vanderbilt.edu

Supplemental Figures

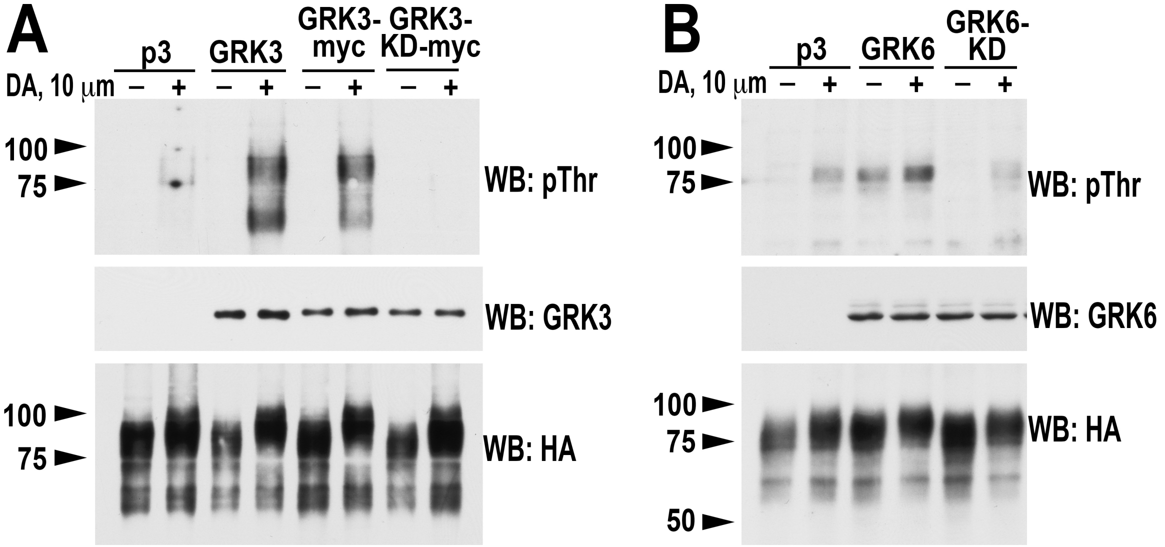

<u>Figure S1.</u> **Mutant GRK3 and GRK6 are devoid of kinase activity**. (**A**) GRK3 with myc tag at C-terminus is fully functional, and GRK2-K220R mutant lacks enzymatic activity. The kinase activity of the wild type myc-tagged GRK3 and GRK3-K220R-myc mutant was tested using human D1 dopamine receptor (D1R) as a substrate. HEK293-FT cells were transfected with D1R with N-terminal triple HA tag together with empty pcDNA3 plasmid (p3), untagged wild type GRK3, myc-tagged wild type GRK3, or myc-tagged GRK3-K220R mutant. Cells were stimulated for 10 min with 10 μM DA/1mM ascorbic acid or with vehicle. D1R was immunoprecipitated on anti-HA antibody coupled with protein-G agarose and blotted for phosphor-threonine. Upper panel shows the level of D1R phosphorylated at threonines. Untagged and myc-tagged GRK3 show similar level of D1R phosphorylation, whereas GRK3-R220R shows no detectable phosphorylation. Middle panel – expression of GRK3 constructs detected with anti-GRK3 antibody. Lower panel – the level of immunoprecipitated D1R detected with anti-HA antibody. (**B**) The activity of wild type GRK6 and GRK6-K215,216M was measured in HEK293-FT cells with human D1R as a substrate as described above. Wild type GRK6 shows agonist-independent and agonist-induced phosphorylation of D1R, whereas GRK6-K215,216M lacks either. Middle panel – GRK6 expression detected with anti-GRK6 antibody. Lower panel - the level of immunoprecipitated D1R detected with anti-HA antibody.

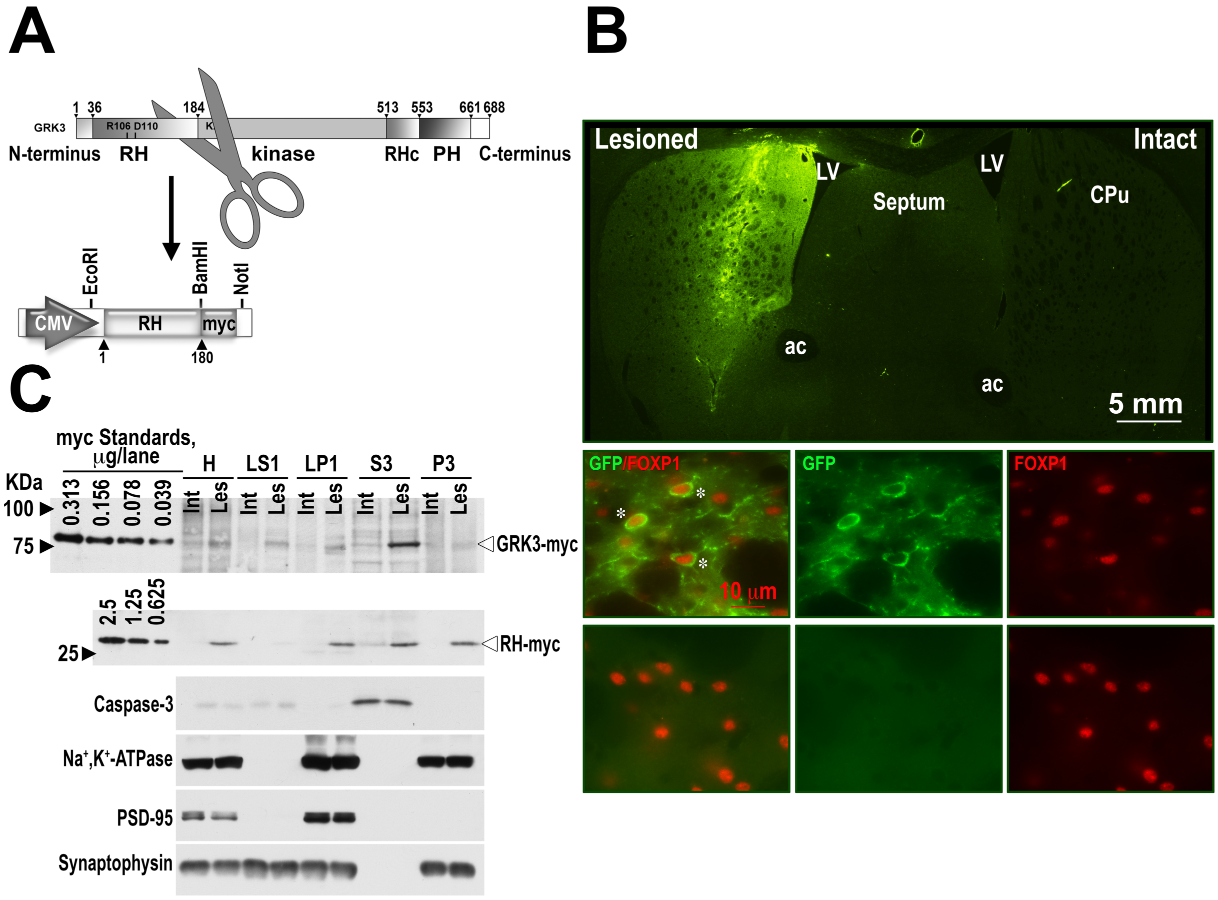

<u>Figure S2.</u> The lentivirally-transferred isolated RH domain is expressed in striatal neurons and has subcellular distribution similar to full-length GRK3. (**A**) Domain structure of rat GRK3 and construction of the lentivirus containing isolated RH of GRK3 [1]. Residues 1 to 180 containing N-terminal helix a0 and N-terminal helices 1-9 of RH were subcloned into the lentiviral vector as shown. RH – RGS homology domain; RHc – C-terminal helices 10 and 11 of the RH; kinase – kinase domain; PH – pleckstrin homology domain; (**B**) The expression of RH in medium spiny striatal neurons was detected by immunohistochemistry to co-cistronically expressed GFP (green) with anti-GFP antibody combined with the staining for FOXP1 (red) as described in Methods. (**C**) Subcellular localization of isolated RH with myc N-terminal tag was compared with that of myc-tagged wild type GRK3. Rats were injected into the striatum with lentiviruses encoding either RH of GRK3, striata were isolated, and subcellular fractionation was performed as previously described [2, 3]. Caspase-3 was used as the marker for cytosol fraction (S3); Na^+,K^+,ATPase – for light membrane fraction (P3); PSD95 – for synaptic membrane fraction (LP1); synaptophysin is detected in P3, LP1, and crude synaptic vesicle fraction (LS1); H – homogenate. Left lanes in GRK3-myc and RH-myc blots show standards in serial dilutions obtained by infecting HEK293 cells with respective lentiviruses.

Supplemental Methods

In cell receptor phosphorylation

Receptor and GRK constructs. Expression constructs for D1 dopamine (D1R) and D2 dopamine (D2R) receptors N-terminally tagged with triple HA were described previously (25,26). Full-length WT untagged GRK3 and GRK6 (kindly provided by Dr. J. L. Benovic, Thomas Jefferson University) in pcDNA3 were used. The mutations to render GRK3 and GRK6 kinase-dead (GRK3-K220R and GRK6-K215,216M) were introduced by PCR and confirmed by dideoxy-sequencing.

Cell culture, transfection, and stimulation. HEK-293-FT cells were maintained in Dulbecco's modified Eagle medium supplemented with 10% FBS and 1% penicillin-streptomycin in a humidified incubator at 37°C and 5% CO2. Lipofectamin2000 (Invitrogen, Carlsbad, CA) (1:2.5 DNA:lipid) in Opti-MEM was used to transfect cells. DNA amounts in each transfection were kept constant by the addition of empty vector, where necessary. All experiments were conducted 48 h post-transfection.

Antibodies. Anti-HA antibody for immunoprecipitation was from Roche Diagnostics (Indianapolis, IN); anti-phospho-threonine antibodies were from Cell Signaling Technology (Beverly, MA) and anti-phospho-serine from Invitrogen (Carlsbad, CA).

Immunoprecipitation. HEK292FT cells co-transfected with indicated receptor and GRK clones were stimulated with dopamine (10 μM) for 10 min at 37°C (control cells were exposed to vehicle for the same time), scraped off plates, collected by centrifugation in phosphate-buffered saline and resuspended in the immunoprecipitation buffer (IPB) containing 50 mM Tris-HCl, 2 mM EDTA, 250 mM NaCl, 10% (v/v) glycerol, 0.5% NP-40, 20 mM NaF, 1 mM sodium orthovanadate, and 10 mM N-ethylmaleimide. Benzamidine (2 mM final concentration) and phenylmethylsulfonyl fluoride (1 mM) were added immediately before use. Cells were lysed at 4°C for 1 h and centrifuged to remove the debris. The supernatant was pre-cleared by incubating with 25-30 μl of Protein G Agarose for 1 h at 4°C. Receptors were then immunoprecipitated by incubating the supernatant overnight at 4°C with anti-HA antibody (1-2 μg per 60 mm dish) and 20-25 μl of Protein G agarose. Beads were washed three times with IPB, and bound proteins were eluted by boiling in Laemmli SDS buffer for 5 min.

Western blotting. The proteins were analyzed by reducing SDS-PAGE and Western blotting onto Immobilon-P (Millipore, Bedford, MA, USA) membrane. The membrane was blocked with 5% non-fat dry milk in TBS at room temperature for 1 h, then incubated in TBS supplemented with 0.1% Triton X-100 and 1% BSA and appropriate primary antibody overnight at 4°C. Blots were incubated with secondary antibodies coupled with horseradish peroxidase (Jackson Immunoresearch Laboratories, West Grove, PA, USA) for 1 h at room temperature, and bands were visualized by SuperSignal enhanced chemiluminescence reagent (Pierce, Rockford, IL, USA).

Fractionation.

Rats were injected with lentiviruses encoding either wild type GRK3-myc or RH-myc. The striatal tissue was isolated as fractioned as described previously [2, 4]. Briefly, approximately 12 mg of fresh striatum tissue was homogenized in 10 volume of ice-cold HEPES-buffered sucrose (0.32 M sucrose, 4mM HEPES pH7.4, 1mM EGTA) containing protease inhibitor cocktail (Sigma-Aldrich, St.Louis. MO) in glass-teflon homogenizer. Homogenate (H) was centrifuged at 1000xg for 10 min at 4°C. Supernatant (S1) was centrifuged at 10,000xg for 15 min to obtain crude synaptosomal fraction (P2) and supernatant (S2). The synaptosomal pellet was lysed by hypo-osmotic shock in 9 volume of ice cold HEPES-buffer with protease inhibitor cocktail for 30 min. The lysate was centrifuged at 25,000g for 20 min at 4°C to obtain synaptosomal membrane fraction (LP1) and crude synaptic vesicle fraction (LS1). Supernatant (S2) was centrifuged at 165,000g for 2 hours to obtain cytosolic fraction (S3) and light membrane fraction (P3). Protein

concentration in the samples was measured with Bradford reagent (Bio-Rad, Hercules, CA). Samples were then precipitated with 90% (v/v) methanol. The protein was pelleted by centrifugation [10,000xg, 10 min at room temperature (RT)], washed with 1 ml of 90% methanol, dried, and dissolved in sodium dodecyl sulfate sample buffer at the final concentration of 0.5 mg/ml. Caspase-3, Na^+,K^+-ATPase, and PSD-95 were used as markers for the cytosol, light membrane (P3) and synaptic membrane (LP1) fractions, respectively.

References

1. Lodowski, D.T., Tesmer, V.M., Benovic, J.L. & Tesmer, J.J. The structure of G protein-coupled receptor kinase (GRK)-6 defines a second lineage of GRKs. *J Biol Chem.* **281**, 16785-16793 (2006).
2. Ahmed, M.R., Bychkov, E.R., Gurevich, V.V., Benovic, J.L. & Gurevich, E.V. Altered expression and subcellular distribution of GRK subtypes in the dopamine-depleted rat basal ganglia is not normalized by L-DOPA treatment *J Neurochem* **104**, 699-711 (2007).
3. Bychkov, E., Ahmed, M.R., Gurevich, V.V., Benovic, J.L. & Gurevich, E.V. Reduced expression of G protein-coupled receptor kinases in schizophrenia but not in schizoaffective disorder. *Neurobiol Dis* **44**, 248-258 (2011).
4. Ahmed, M.R. *et al.* Lentiviral overexpression of GRK6 alleviates L-dopa-induced dyskinesia in experimental Parkinson's disease. *Sci Transl Med* **2**, 28ra28 (2010).
